# Changes in tumor-antigen expression profile as human small-cell lung cancers progress

**DOI:** 10.7497/j.issn.2095-3941.2015.0027

**Published:** 2015-06

**Authors:** Li-Sheng Ge, Neil T. Hoa, Nils Lambrecht, Maria Dacosta-Iyer, Yi Ouyang, Amir Abolhoda, Martin R. Jadus

**Affiliations:** ^1^Research Service, ^2^Pathology and Laboratory Medicine Service, VA Long Beach Healthcare System, Long Beach, CA 90822, USA; ^3^Pathology and Laboratory Medicine, University of California, Irvine, CA 92697, USA; ^4^Surgical Health Care Group, Veterans Affairs Medical Center, Long Beach, CA 90822, USA; ^5^Chao Family Comprehensive Cancer Center, UC, Irvine School of Medicine, University of California, Irvine, Orange, CA 92868, USA

**Keywords:** Small-cell lung cancer (SCLC), glioma big potassium (gBK) ion channel, tumor antigens, immunoprevention, real-time polymerase chain reaction, T-lymphocytes

## Abstract

**Objective:**

Our group has previously observed that in patients with small-cell lung cancers (SCLCs), the expression of a tumor antigen, glioma big potassium (gBK) ion channel, is higher at the time of death than when the cancer is first treated by surgical resection. This study aimed to determine whether this dichotomy was common in other potential lung tumor antigens by examining the same patient samples using our more extensive profile analysis of tumor-antigen precursor protein (TAPP). We then tested the hypothesis that therapeutic intervention may inadvertently cause this increased gBK production.

**Methods:**

SCLC samples (eight surgical resections and three autopsy samples) and three control lungs were examined by quantitative real-time polymerase chain reaction for 42 potential TAPPs that represent potential T-cell-mediated immunological targets.

**Results:**

Twenty-two TAPP mRNAs displayed the same profile as gBK, i.e., more mRNAs were expressed at autopsy than in their surgical counterparts. B-cyclin and mouse double minute 2, human homolog of P53-binding protein were elevated in both autopsy and surgical specimens above the normal-lung controls. When HTB119 cells were incubated with doxorubicin, gBK was strongly induced, as confirmed by intracellular flow cytometry with a gBK-specific antibody.

**Conclusion:**

Our findings suggested that more immunological targets became available as the tumor responded to chemotherapy and proceeded toward its terminal stages.

## Introduction

Immunotherapy significantly affects the treatment of established human cancers. Dendritic cell (DC)-based immunotherapies use the patient’s own DCs that are fed with tumor extracts or antigenic peptides and are infused back into the patient. These antigen-loaded DCs then migrate to the lymph nodes and activate the host’s T-cells. These stimulated endogenous T-effector cells in turn seek out and kill the remaining tumor cells. In glioblastoma multiforme (GBM), this therapy has been proven effective against the “mesenchymal” subtype of GBM^1^. Positive responses have been observed for DC stimulated with the “Provenge” fusion molecule, i.e., the survival of patient with castrate resistant prostate cancer increases by 4 months^2^. Lung-cancer vaccines including those that use DC pulsed with antigenic peptides or killed whole cells are being developed and have been reviewed by Jadus *et al*.^3^. Antibodies toward  so-called “check-point inhibitory” pathways such as programmed cell death-1, programmed cell death-1 ligand, and cytotoxic T-lymphocyte antigen-4 likewise affect patient survival in various cancer types, including non-small cell lung cancers (NSCLCs)^4^^-^^6^. These inhibitory molecules are expressed on regulatory T-cells (Treg) and tumor cells, effectively suppressing antitumor immune functions. In clinical trials using these check-point inhibitory antibodies, only about 25%-30% of cancer patients are successfully treated^6^, leaving plenty of room for improvement. Apart from understanding how tumors can inhibit the immune system, the identification of tumor antigens that can be used as potential vaccines is also important to prevent future tumor growth. By stimulating immune responses toward the cancer, more activated T-cells can be directed toward the tumor, which can eliminate tumor cells that are inaccessible to surgery or radiation. DCs loaded with tumor antigens can be easily merged with check-point inhibitory strategies to produce even better clinical outcomes.

Previously, our group has worked with the glioma big potassium (gBK) ion channel^7^^,^^8^. This ion-channel variant has a 32 amino-acid insert found within the intracellular region of this BKα chain. This ion channel, initially cloned from human D54 glioma cells^9^ (hence its initial descriptive name), is found within a wide variety of cancer types^7^^-^^9^ but not within non-tumorous lymphocytes, fibroblasts, or human embryonic kidney cells. Ion channels including potassium, sodium, and chloride ion channels play important roles in tumor-cell migration^10^^,^^11^. BK channels are believed to play a role in glioma-cell migration^10^^,^^11^. Both gliomas and SCLCs are invasive cancers and could thus have similar migratory properties using these BK and gBK channels. BK channels, and probably gBK, are mechanosensitive ion channels, meaning that these channels are activated when the membrane is physically stretched^12^. Consequently, once internal K^+^ cations are released, a positive feedback loop starts this infiltrative process and continues as the cell moves. X-ray irradiation of human T98G and U87 glioma cells immediately activates their BK channels and initially increases the mobility of these cells than their non-irradiated counterparts^13^. SCLCs favorably respond to radiation at the beginning^14^, but then the cancer returns at another anatomic site. Thus, BK ion channels may drive the invasion/metastatic processes of cancer cells as a consequence of therapeutic ionizing irradiation. When our group has investigated gBK with SCLC, we have discovered that SCLC autopsy specimens contain higher gBK mRNA levels than GBM autopsy material^8^. We have failed to see any up-regulation of the lung specific transcription factor Sox11 within the studied SCLC autopsy cases. Thus, this phenomenon is unlikely to be an artifact of the patient death process or simple RNA degradation.

In the present work, we analyzed eight surgical samples from SCLC patients taken early in their treatment and found that these samples possessed minimal gBK mRNA. To determine whether this gBK dichotomy was an anomaly, we examined 42 other tumor antigens known to elicit T-cell-mediated responses. Twenty-two tumor-antigen precursor proteins (TAPPs) followed the same pattern as gBK. Two TAPPs, B-cyclin and mouse double minute 2, human homolog of P53-binding protein (MDM2), were elevated in both SCLC subsets studied. The remaining 18 TAPP mRNAs failed to show any significant expression within the SCLC specimens. We concluded earlier that this antigen expression profile may be an example of tumor-antigen selection or tumor-cell clonal expansion. However the possibility exists that tumor antigen evolution may drive this antigen progression as a result of active therapeutic intervention. We used an HTB119 cell line that had not been previously exposed to any therapeutic intervention before this cell line was established. We showed an induction of gBK in response to doxorubicin exposure; thus, gBK may be a prototypical tumor antigen that can be stimulated by therapeutic intervention. This discovery can serve as a basis for the development of rational immunological interventions for antigens before the cancer expresses them. Therefore, immunoprevention can be considered to prevent the terminal stages of SCLC.

## Materials and methods

### Cell culture

HTB119 (NCI-H69) and H1436 (NCI-H1436) SCLC cell lines were purchased from American Type Culture Collection (Manassas, VA, USA) and was cultured as described elsewhere^8^.

### Chemicals

All chemotherapeutic agents were purchased from Sigma Chemical Corporation (St. Louis, MO, USA).

### Lung tumor tissue

After an IRB expedited review was granted for this project, we collected either autopsy material or excess surgical material not needed for diagnostic purposes. We collected three autopsy samples from our SCLC cases, along with three lung samples taken from other autopsies, which did not have lung cancer. We purchased eight SCLC surgical cases from Oncomatrix (San Diego, CA, USA), who obtained their tissue from Eastern Europe where surgical resections of SCLC patients are still performed.

### Quantitative reverse-transcriptase real-time polymerase chain reaction (qRT-PCR)

We used the same techniques and primers as those previously described^15^^-^^17^. We also designed primers that are reported to be expressed or over-expressed within lung cancers and to be able to elicit human immune responses^18^. These TAPPs included calgranulin forward: 5'-GCCACCACCATAAGCCAG-3'; reverse: 5'-ACCATGACTGTGGCCGTG-3'; CTL-recognized and on melanoma (CAMEL) forward: 5'-CGCTTCTGCGCAGGATGG-3'; reverse: 5'- CCATGGGCGACGAGAAAG-3'; cyclophilin B forward: 5'-CTCTTCCGGCCTCAGCTG-3'; reverse: 5'-AAGACGGACCCCGCGATG-3'; livin forward: 5'-CGTGGTGGGTTCTTGAGC-3'; reverse: 5'-CACGGCACAAAGACGATG-3'; MDM2 forward: 5'-ATGATCCCCGAGGCCCAG-3'; reverse: 5'-CCGGGGTTTTCGCGCTTG-3'; parathyroid hormone-related protein (PTH-rP) forward: 5'-CCAAGGACATATTGCAGG-3'; reverse: 5'-GCAGTTTCATAGAGCAATGG-3'; trio guanine nucleotide exchange factor and regulates actin (TARA) forward: 5'-GCCATGACGCCCGATCTG-3'; reverse: 5'-AGGTGGT-GGTGAGCGAGG-3'; transthyretin (TTR) forward: 5'-GTCTGAGGCTGGCCCTAC-3'; reverse: 5'-ACGGCCACATTGATGGCAG-3'; X antigen family, member 1B (Xage-1B) forward: 5'-TTCGCCAGTGTGGGGAAC-3'; reverse: 5'-CCGCCGTGTCTCAGTAGC-3'. Samples were run in quadruplicate, and a reaction without cDNA established a baseline fluorescence level with 18S RNA. Fluorescent signal versus cycle number was plotted. Threshold cycle (C_t_) was defined as the cycle number where a fluorescence signal could be reliably detected above background fluorescence. Each PCR run also included non-template controls containing all reagents except cDNA. After cycling, a melting curve was produced by slow denaturation of PCR end products to validate amplification specificity. The relative quantification of expression of any given gene was determined by the 2^-ΔCt^ method^19^. mRNA expression was considered as a strongly positive signal when ΔC_t_ = [6-14], as a moderately positive signal when ΔC_t_ = [15-20], as a weak positive signal when ΔC_t_ = [21-24], and as a negative signal when ΔC_t_ >24 (the line displayed on the various panels). To statistically analyze the data, an arbitrary ΔC_t_ score of 25 was assigned to specimens in which a signal for the TAPP was not detected. Based on our previous qRT-PCR experience, a cutoff point of 24 was set for the ΔC_t_ value that was biologically relevant. Cell lines having an mRNA score below this value routinely lacked protein for a given gene detected by antibody-based methods^15^. Data from each tumor sample was grouped according to tumor type and plotted. Statistical differences between normal-lung and SCLC samples were calculated using unpaired students *t*-tests, with *P*<0.05 considered significant.

### Immunofluorescent microscopy

Three archival SCLC paraffin blocks collected from patients who died from their cancers with distant metastases were randomly chosen. Recut slides were first deparaffinized and treated with an antigen-retrieval reagent. These slides were immunostained using either non-immunized IgGs from different species, the mouse monoclonal anti-tyrosinase-related protein (Trp)-75 (also called Trp-1), goat anti-melanoma antigen (Mage)-1 polyclonal antibody [Lab Vision/Neomarkers (Fremont, CA, USA), or rabbit anti-telomerase reverse transcriptase (hTert) antibody (Santa Cruz. Biotech, Santa Cruz, CA, USA)]. The slides were washed and stained with the appropriate FITC-conjugated secondary antibodies (Vector Labs, Burlingame, CA, USA). Representative fluorescence images were captured using a Nikon Eclipse E600 fluorescence microscope with RTKE Spot Camera and Spot software.

### Intracellular flow cytometry

Exponentially growing SCLC cells were fixed in Santa Cruz Biotechnology fixative, washed, and permeabilized using Santa Cruz Biotechnology. Afterwards, the cells were incubated with the primary antibody. The goat polyclonal antibody directed toward human gBK was made by GenScript Inc. (Piscataway, NJ, USA)^7^^,^^8^. A total of 10,000 cells were evaluated with a Bectin-Dickinson FACS Profile flow cytometer. The Kolmogorov-Smirnov statistics test was used to show the significance of differences in antibody staining profiles at *P*<0.05 level.

## Results

### Enhanced TAPP mRNA expression occurred only within SCLC autopsy specimens

Our initial work dealt with cancer cells and the gBK ion channel^7^^,^^8^. We unexpectedly found that resected samples of SCLC (*n*=8) taken early during disease treatment possessed little gBK mRNA, as detected by qRT-PCR. However, at the time of death of three SCLC patients, gBK was highly expressed in these specimens. SCLC is derived from neuro-endocrine precursor cells (Kulchitsky cells)^20^ and is an infiltrative tumor^14^ like GBM, so gBK expression could indicate the invasive nature common to both cancers. We used our TAPP primer bank^15^^,^^16^ to determine whether this dichotomy was a common or a novel phenomenon. We used some “universal” (e.g., B-cyclin, hTert, her2/neu, Mage, and MRP3) and “lung-specific” (e.g., CAMEL, calgranulin, and Xage-1B)^18^ antigens to address this issue.

**Figure 1:**
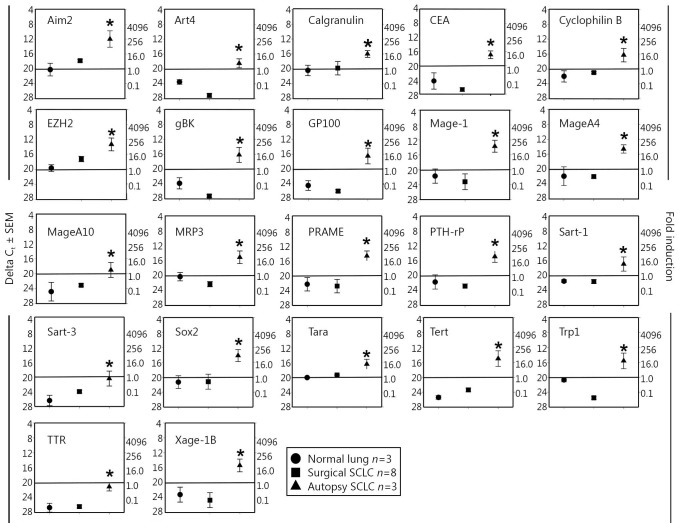
Twenty-two TAPPs are expressed only within SCLC autopsy samples. Eight surgical resections of SCLC (square symbols), 3 SCLC autopsy samples (triangle symbols) and 3 control lungs samples (circle symbols) from autopsy patients who did not have any cancer were examined for these 22 TAPPs. Asterisks denote significant statistical differences (*P*<0.05) between the SCLC autopsy and normal lung autopsy values. The data on gBK was previously published in reference 8 and is shown here for comparison purposes with the other antigens.

We collected three control lung samples that did not have cancer, and together with the previously used eight surgically resected SCLC specimens and three SCLC autopsy samples^8^, we subjected these RNA samples to qRT-PCR analysis. Results showed that for 22 TAPPs, the expression of these antigens was significantly elevated at the time of autopsy, and no or very low expression was observed when the SCLC surgical samples were compared with non-cancerous lung tissue (**Figure 1**). These potential antigens included the following: antigen isolated from immunoselected melanoma-2, Aim2, antigen recognized by T-cells (ART)-4, calgranulin, carcinoembryonic antigen, cyclophilin B, polycomb group protein enhancer of zeste homolog 2 (EZH2), gBK, melanoma glycoprotein-100 (GP100; also called Pmel 17), livin, Mage-1, Mage-A4, Mage-A10, multidrug-resistance protein-3 (MRP-3), preferentially expressed antigen in melanoma (PRAME), PTH-rP, squamous cell carcinoma ARTs (Sart)-1, Sart-3, the transcription factor Sox2, TARA, hTert, Trp-1, TTR, and Xage-1B. All these TAPP mRNAs were absent from SCLC surgical samples but present in autopsy samples.

### Two TAPPs were elevated in both surgical and SCLC autopsy samples

**Figure 2:**
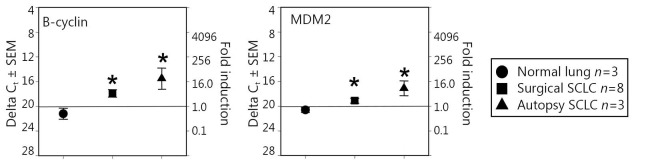
Significant TAPP expression in both surgical and autopsy SCLC. The same RNA preparations used in Figure 1 were examined for B-cyclin and MDM2. In both experiments the mRNA levels displayed significant differences (*P*<0.05) between the normal lung mRNA when compared to the SCLC specimens.

We found that two putative precursor antigens (B-cyclin and MDM2) were significantly elevated both in surgical samples and autopsy samples compared with normal-lung autopsy specimens (**Figure 2**). The surgical samples had an intermediate value, whereas the autopsy samples had higher amounts of mRNA. Thus, not all antigens displayed this all-or-none effect, suggesting that an intermediate level of TAPP can exist in SCLC surgical samples.

### Immunofluorescence microscopy confirmed that Tert, Mage-1, and Trp-1 TAPPs were present in SCLC autopsy specimens

**Figure 3:**
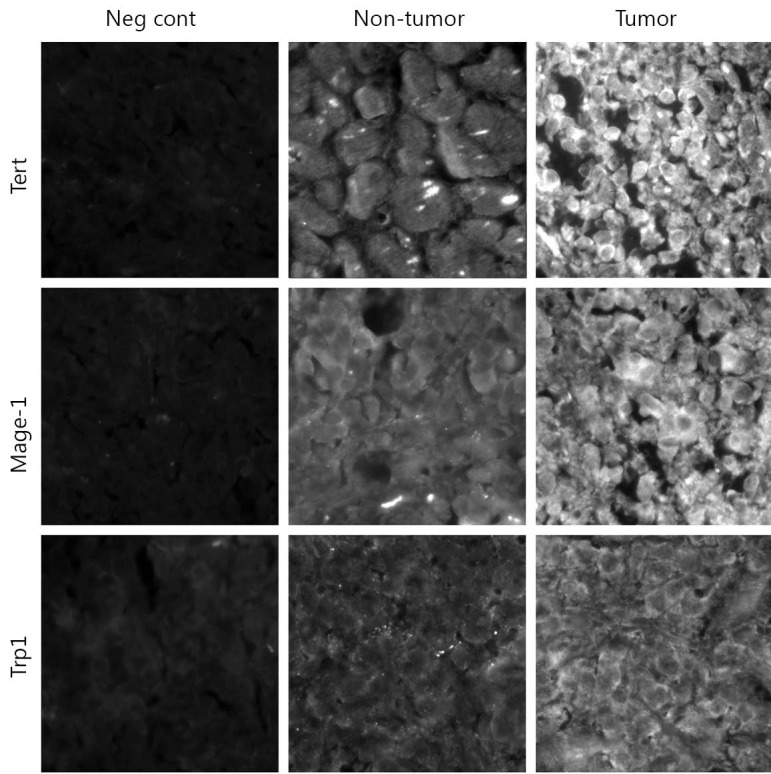
Immunofluorescence microscopy shows 3 TAPPs are present within SCLC specimens taken at autopsy. Three SCLC specimens were examined for expression of h-Tert, Mage-1, and Trp-1 by using antibodies directed towards these 3 TAPPs.

We confirmed the presence of three TAPPs at the protein level within SCLC samples by using antibodies directed against Tert, Mage-1, and Trp-1 proteins. Three archival SCLC histological samples with confirmed distant metastases collected from SCLC patients at the time of their deaths were stained with their respective antibodies, washed, and stained with an FITC-conjugated secondary antibody. All three SCLC samples showed tumors positively stained for these three TAPPs. The representative staining patterns shown in **Figure 3** were those of samples collected from a metastatic lesion from the left ventricle. The first column shows the negative controls in which non-immunized IgGs were applied with the appropriate secondary antibody. The middle column shows a non-tumorous region of the left ventricle, and the right column shows the nest of SCLC cells. The top, middle, and bottom rows show Tert, Mage-1, and Trp-1 stainings, respectively. All SCLC cells stained strongly positive. The non-tumorous lung tissue was negative or weakly positive, as proven by their lowered green fluorescence; this internal control showed that the antibody did not stain every cell within the lung.

### Not all TAPP mRNA were found within autopsy samples

**Figure 4:**
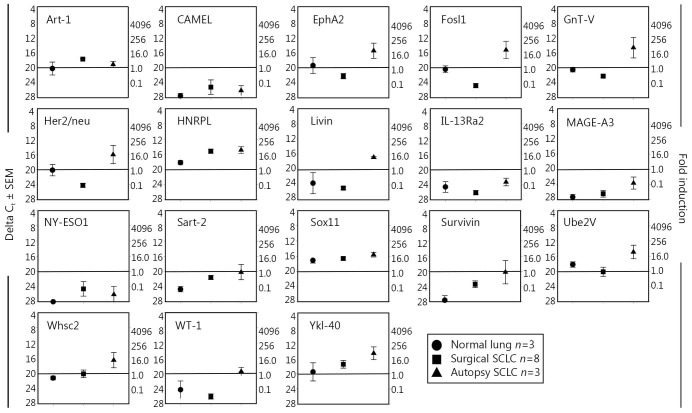
TAPP expression does not show a differential pattern within SCLC tumors or normal lung tissue. The same preparations of RNA used in Figures 1 and 2 were also analyzed with these 18 different primer sets. The data from Sox11 was used in a previous paper^8^.

A third TAPP mRNA profile failed to show any TAPP expression above control autopsy lung tissue levels (**Figure 4**). These 18 putative antigens included the following: ART-1, CAMEL, interleukin-13 receptor-α2, Mage-A3, New York esophageal-1 antigen, NY-Eso1, Sox11, and Trp-2. Twelve TAPPs [EphA2, Fos-related antigen-1 (also called Fosl1)], β1,6-N-acetylglucosaminyltransferase-V (GnT-V), her2/neu, high nuclear riboprotein-L (HNRPL), livin, Sart-2, survivin, Wolf-Hirschhorn syndrome candidate 2 protein (Whsc2), Wilms tumor antigen-1 (Wt-1), Ube2V, and chitinase 3-like 1 [cartilage glycoprotein-39; also called Tyr-Lys-Leu containing protein-40 (Ykl-40)]. They had overall trends toward being expressed but failed to show statistical significance perhaps because of the low numbers of SCLC autopsy samples that were tested.

### Doxorubicin can enhance gBK transcription in the HTB-119 SCLC cell line

**Figure 5:**
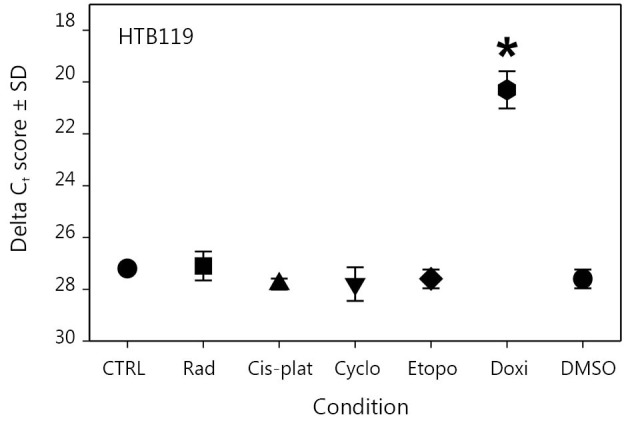
gBK expression is induced in HTB119 by doxorubicin. HTB119 cells were cultured for two weeks under the various conditions. The cells were irradiated at Day 0 with 1,000 rad and then cultured. Cells were cultured with cis-platinum, cyclophosphamide, Etoposide or doxorubicin. The cells were lysed, the RNA was isolated and then analyzed for gBK or 18S RNA by qRT-PCR.

We used an HTB119 cell line established from an SCLC patient who had not been subjected to any therapy^20^^,^^21^. This cell line, which expressed minimal gBK protein, represented an initial SCLC case that was not selected because of any clinical intervention^8^. We collected these HTB119 cells and treated them for 2 weeks with various chemotherapeutic drugs that had been used to treat SCLC^22^. These cells were examined for gBK expression by qRT-PCR. **Figure 5** shows the results of this experiment. Sub-lethal radiation (1,000 rad) and the drugs cisplatinum (50.0 µM), cyclophosphamide (2.5 µM), etoposide (50.0 µM), and doxorubicin (5.0 µM) were examined for their effect on gBK mRNA levels. Cis-platinum, cyclophosphamide, and radiation failed to enhance gBK expression, whereas etoposide gave a slight elevation of gBK (1.5-fold), but this was not considered significantly different from the untreated control values (*P*=0.084). Doxorubicin displayed an elevated level of gBK transcription (115-fold). This mRNA level proved to be significant between the untreated control SCLC and those treated with doxorubicin (*P*=0.0001).

**Figure 6:**
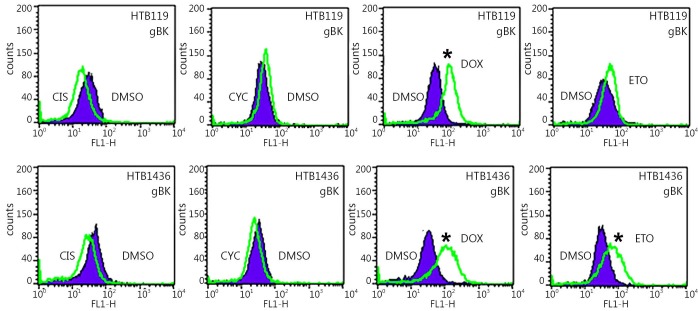
gBK protein is induced with HTB119 and H1436 by doxorubicin. The exact same chemotherapeutic conditions from (Figure 5) were repeated using both HTB119 and H1436 SCLC cells. After 2 weeks of incubation, the cells were fixed, permeabilized and stained for gBK using the goat anti-human gBK antibody. After washing, they were stained with a mouse anti-goat IgG-FITC antibody. Subsequently these washed cells were analyzed by flow cytometry. Ten thousand cells were examined. The shaded area represents the fluorescence of the DMSO-treated controls stained for gBK, while the open area is the profile of the drug treated cells stained for gBK. CIS is Cis-platinum treated cells, CYC is cyclophosphamide treated cells, DOX is doxorubicin treated cells and ETO is Etoposide treated cell.

We showed that elevated gBK mRNA expression was translated into protein by repeating the previous experiment and performing intracellular flow cytometry with the polyclonal antibody to gBK. **Figure 6** shows that doxorubicin induced gBK expression in both HTB119 (top row) and another SCLC cell line, H1436 (bottom row). Etoposide significantly induced gBK protein expression in H1436 cells.

## Discussion

The incidence of lung cancer is now declining in the USA as a result of active anti-smoking campaigns. However, in many developing countries, smoking and lung cancer rates are rising. SCLC accounts for about 15% of all diagnosed lung cancers, but very little clinical progress has been made in treating this form of lung cancer^23^. The 5-year survival of patients with this cancer is still dismal because of the cancer’s invasive/metastatic behavior. Promising clinical results occur when antibodies to “inhibitory checkpoints” are given to patients with various solid-organ cancers^4^^-^^6^, including NSCLCs. Understanding of the mechanisms by which host immunity can be augmented is important, but so is identifying antigens that can stimulate T-cell immune responses. Currently, the known antigens for SCLCs are few. Accordingly, this study was conducted to identify putative antigens with possible therapeutic use for SCLC patients. Once SCLC patients were vaccinated for the right antigens, these patients had more activated T-cells circulating in them, and the use of check-point inhibitory antibodies further enhanced the effectiveness of these T-cells.

The gBK ion channel was initially recognized to be highly expressed only within SCLC autopsy samples, whereas very little gBK was detected in early-stage SCLC. What process led to our phenomenon still processes led to our phenomenon remains to be investigated. We expanded our prior work and studied a large panel of potential TAPPs to determine whether this dichotomy of expression was applicable to other tumor antigens. Indeed, we found that most TAPPs were elevated within SCLC autopsy samples. Many of these antigens were of a universal nature such as B-cyclin, Tert, and MRP-3, which allowed cancer cells to grow, replicate without chromosome shortening, and resist chemotherapy, respectively. Thus, the expression of these antigens did make biological sense. We confirmed that three TAPPs (Trp-1, Mage-1, and h-Tert) were elevated within three SCLC autopsy samples and were translated into protein as detected by antibody staining (**Figure 3**). Many of these up-regulated TAPPs are common in gliomas and other brain cancers^15^^,^^16^, such as Aim2, EZH2, GP100, Mage-1, MageA4, MageA10, Sart-1, -3, Trp-1, and Sox2 (**Figure 1**). SCLCs are derived from neuro-endocrine precursor cells, the so-called Kulchitsky cells^20^^,^^21^, which explains the shared antigen profile of SCLC with human gliomas. However, not every glioma specific TAPP was found to be elevated in these SCLC samples (**Figure 3**), e.g., IL-13Rα2. Thus, some differential specificity between SCLC and gliomas remained.

Twelve TAPPs (EphA2, Fosl1, GnT-V, her2/neu, HNRPL, livin, Sart-2, survivin, Ube2V, Whsc2, Wt-1, and Ykl-40) had overall trends toward being different but failed to show statistical significance because of the low number of SCLC or control lung-autopsy samples tested to date, along with the higher standard error of means within these two groups. We began this study about 6 years ago and have collected only three SCLC autopsy cases to date. If this study was conducted about 20-30 years ago, more lung-cancer autopsies would have been easily collected. Nevertheless, we did achieve statistical significance for a number of TAPPs (**Figures 1****,****2**), but not for the 12 TAPPs that were trending in that direction (**Figure 3**). This research indicated that autopsies can still provide valuable insight into cancer biology and should still be encouraged.

We initially believed that this progression of tumor antigens within SCLC may represent only the natural progression of this cancer. SCLC patients are almost always treated with either radiation or chemotherapeutic drugs. Previously, SCLCs have been treated with cis-platinum, cyclophosphamide, doxorubicin, or etoposide^22^. In the present study, we used the SCLC cell line HTB119 and showed that these cells displayed some gBK, but not as much as H1436 cells isolated after a patient received medical therapy. HTB119 cells were collected from a patient who did not receive any therapy^23^^,^^24^; hence, this tumor represented an early-stage SCLC. When doxorubicin was cultured with these HTB119 cells, gBK was reproducibly increased (**Figures 5****,****6**); when we cultured H1436 SCLC cells with doxorubicin, increased gBK expression occurred. Thus, other SCLC cells can respond in a similar manner, and further investigation on the mechanism of this induction is required. Interestingly, etoposide increased gBK in H1436 cells but not in HTB119 cells. Thus, some chemotherapeutics can drive antigenic expression in SCLC as they proceed toward their final outcome in some unknown manner. This knowledge provides a basis for vaccinating SCLC patients with tumor antigens and perhaps calter the final progression of this cancer through the concept of immunoprevention^25^.

In support of this chemotherapeutic drug-induced gBK production, we observed that human glioma cells behaved the same way using the recently approved drug temozolomide (TMZ). TMZ is an alkylation and methylation drug that improves human GBM survival by 2.5 months^26^. We found that gBK mRNA and protein levels were significantly stimulated by TMZ in human glioma cell lines (TMZ induced the expression of gBK ion channel while inhibiting fascin-1 expression, i.e., possible synergistic enhancements for glioma immunotherapy, manuscript submitted). Thus, gBK induction may be a prototypical antigen mechanism showing that tumor antigen progression can be partly driven by chemotherapy in different cancer patients. This phenomenon of increased TAPP expression has been reported in melanoma cell lines when a BRAF inhibitor (vemurafenib) is administered. As a result of such treatment, GP100, Trp-1, Trp-2, and Mart-1 all increased both at the mRNA and protein levels^27^. Thus, some so-called “specific inhibitory” drugs like doxorubicin, TMZ, and vemurafenib still have off-label effects on the immune system.

In summary, we showed that SCLC autopsy samples highly expressed 24 potential TAPPs that should theoretically elicit human T-cell responses. HTB119 cells that were developed from SCLC before applying any therapy induced gBK to respond to the chemotherapeutic drug doxorubicin. Therefore tumor-antigen expression may be partly driven by chemotherapy and may provide immunological targets for vaccination protocols.
